# *Drosophila* LKB1 is required for the assembly of the polarized actin structure that allows spermatid individualization

**DOI:** 10.1371/journal.pone.0182279

**Published:** 2017-08-02

**Authors:** Jean-Louis Couderc, Graziella Richard, Caroline Vachias, Vincent Mirouse

**Affiliations:** GReD, Université Clermont Auvergne, CNRS, Inserm, Clermont-Ferrand, France; Cardiff University, UNITED KINGDOM

## Abstract

In mammals, a testis-specific isoform of the protein kinase LKB1 is required for spermiogenesis, but its exact function and specificity are not known. Human LKB1 rescues the functions of Drosophila Lkb1 essential for viability, but these males are sterile, revealing a new function for this genes in fly. We also identified a testis-specific transcript generated by an alternative promoter and that only differs by a longer 5’UTR. We show that dLKB1 is required in the germline for the formation of the actin cone, the polarized structure that allows spermatid individualization and cytoplasm excess extrusion during spermiogenesis. Three of the nine LKB1 classical targets in the *Drosophila* genome (AMPK, NUAK and KP78b) are required for proper spermiogenesis, but later than dLKB1. *dLkb1* mutant phenotype is reminiscent of that of myosin V mutants, and both proteins show a dynamic localization profile before actin cone formation. Together, these data highlight a new dLKB1 function and suggest that dLKB1 posttranscriptional regulation in testis and involvement in spermatid morphogenesis are evolutionarily conserved features.

## Introduction

**Fig 1:**
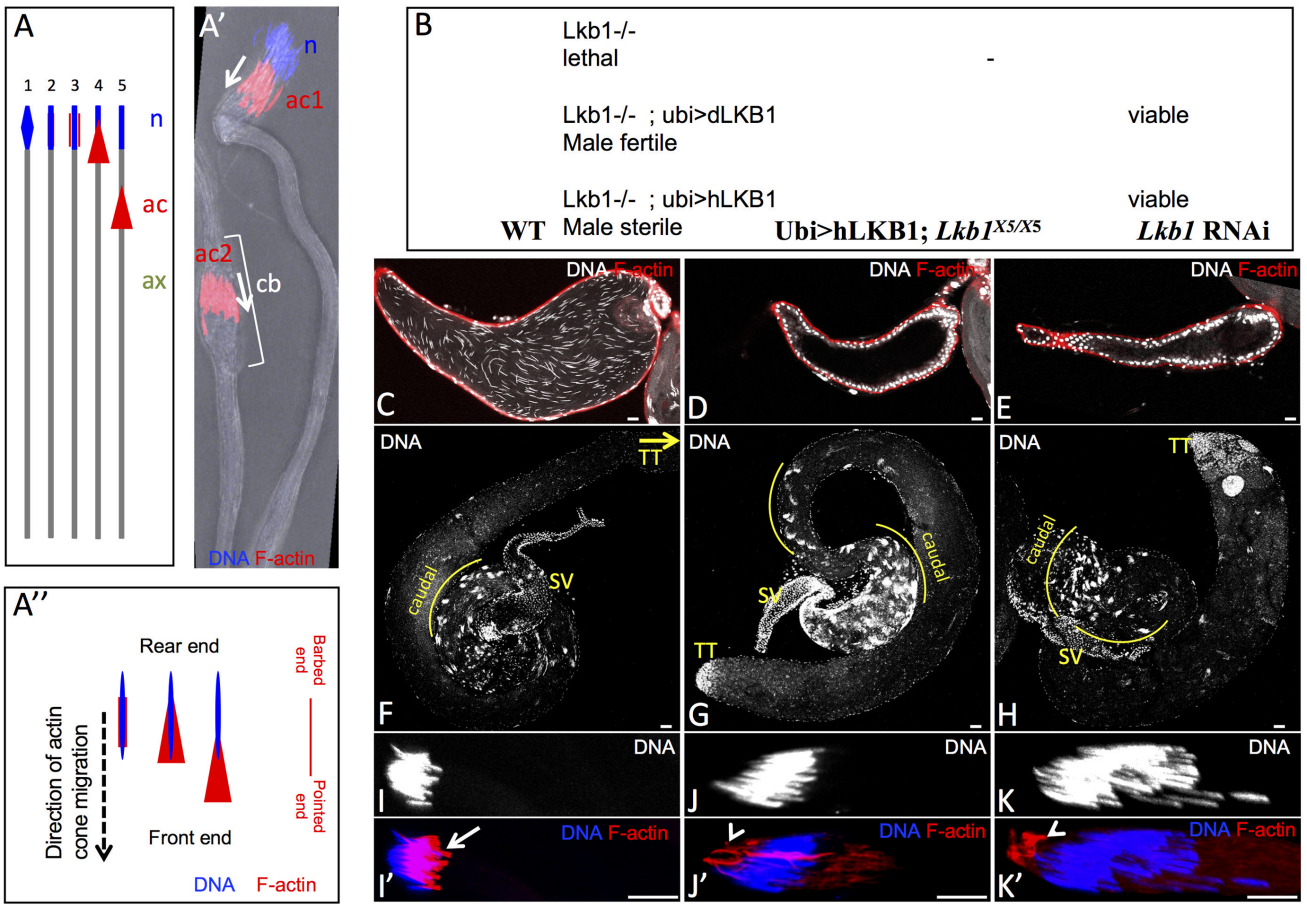
LKB1 is required for male fertility in *Drosophila*. A) Schematic representation of the individualization of a spermatid in *Drosophila*: (1) spermatid nucleus (n; blue) elongation, (2) initiation of actin cone formation, (3) actin cone (ac; red) forming, (4) actin cone begins to migrate, (5) actin cone migrates along the axoneme (ax; green) to ensure spermatid individualization. A’) Actin cone migration during spermiation: an actin cone complex (ac1) starts migrating away from the nucleus (n), and another (ac2) is already far away from the nucleus and pushing ahead the cytoplasm and organelles that form the cystic bulge (cb). A” Schematic representation of actin cones to orientate the direction of migration and to position the rear end and the front end of the actin cones and of the nuclei. The orientation of the actin filament barbed end and pointed end is also indicated. B) Rescue of *Lkb1* lethality with a transgene encoding *Drosophila* Lkb1 (dLkb1) or human LKB1 (hLKB1) driven by the ubiquitin promoter (ubi). Analysis of testes from WT (C, F), hLKB1-rescued adult males (D, G) and from males that express the bam-GAL4–driven RNAi against *dLkb1* (E, H). Seminal vesicles contained free spermatozoa in WT (C), whereas they were empty in hLKB1-rescued (D) and in *dLkb1* RNAi (E) males. (F, G, H) Representative images of the testis caudal region where nuclear bundles of spermatids accumulate before or during individualization. The yellow curves highlight the regions where the spermatid nuclear bundles accumulate. SV, seminal vesicles; TT, testis tip (= rostral region). (I to K’) Testes squashes from WT (I, I’), hLKB1-rescued (J, J’) and *dLkb1* RNAi adult flies (K, K’) showing that actin cone formation (red) in nuclear bundles after elongation of spermatid nuclei is defective in testes from hLKB1-rescued and *dLkb1* RNAi males. Arrow (I’) points to well-ordered arrays of F-actin cones in WT, arrowheads (J’,K’) point to abnormal clumps of actin. DNA (blue) was stained with Hoechst. Scale bars = 10μm.

The *Drosophila* testis is a narrow tube that is closed at the rostral end, where germline stem cells are found, and open at the caudal end, where mature elongated spermatids pass into the seminal vesicle. During spermatogenesis, germ line stem cells divide and produce spermatogonia, each of which undergoes four rounds of mitosis, resulting in cysts of 16 primary spermatocytes. Then, they enter meiosis to give rise to 64 spermatids that are connected by cytoplasmic bridges and encapsulated by two somatic cyst cells [1,2]. In each spermatid, axonemes elongate in a polarized manner, resulting in an extreme intracellular asymmetry. Finally, syncytial spermatids are separated in individual cells with flagella in a process known as sperm individualization that generates 64 mature sperm cells within each cyst. The 64 spermatid nuclei condense and coalesce into a nuclear bundle. They are located at the caudal end of the testis, near the seminal vesicle, and the flagellar tails extend, throughout the testis length, towards its rostral tip. Sperm individualization in *Drosophila* initiates when a polarized actin-based structure, known as actin cone, assembles around each of the elongated spermatid nuclei [3–5] (Fig 1A). The actin cones build a macroscopic structure known as individualization complex [6], and move away concomitantly from the nuclei along the length of the cyst towards the sperm tails. In myosin V mutants fewer actin cones start to form an they failed to extend past the associated nuclei, showing that Myosin V is required for correct actin cone formation [7]. Actin cones have two domains, a rear region of parallel F-actin bundles and a front region of F-actin meshwork (Fig 1A”) [8]. The Arp2/3 complex is required for the formation of the actin meshwork but not for the migration of the cones. Profilin, an other regulator of actin assembly, is required for actin cone movement, Myosin VI is also required for stabilization of the actin cones as they move [8]. F-actin turn-over is observed throughout the cone, with a slightly faster kinetics at the leading edge [9]. As this migration is accompanied by the continuous accumulation of extruded cytoplasmic material around the individualization complex, a voluminous structure called the cystic bulge is created, resulting in the reduction of spermatid cell volume (Fig 1A’) [3–5]. Finally, when the individualization complex reaches the end of the now individualized tails, the cystic bulge turns into a waste bag that is eventually degraded. The separated sperm cells coil up in the cyst and then are released in the testis and freely swimming sperm are transferred and accumulate in the seminal vesicle.

LKB1 is a serine/threonine kinase that regulates multiple cellular processes, such as cell polarity or cell metabolism, by activating AMP-activated protein kinase (AMPK)-related kinase family members [10,11]. LKB1 mutations are associated with many sporadic cancers and also cause Peutz–Jeghers syndrome, an autosomal dominant disease associated with increased risk of cancer [12,13]. LKB1 is evolutionarily conserved and is involved in establishing the polarity of *C*. *elegans* first two asymmetric divisions [14], of *Drosophila* oocytes [15] and of mammalian intestinal cells and neurons [16,17]. Moreover, human and mouse testis-specific LKB1 transcripts have been identified [18,19]. These alternative splice variants are shorter and encode a different C-terminus compared with the longer, widely expressed transcript. Analysis of mice with significantly reduced total LKB1 expression and complete absence of the testis-specific variant revealed very few and abnormal spermatozoa released in seminiferous tubules in its absence, suggesting that the shorter splice variant is essential for fertility [18,20]. However, neither the functional specificity of the testis-specific isoform nor the cellular processes mediated by LKB1 during spermatogenesis have been clearly identified. Moreover, though some LKB1 targets, as AMPK, are involved during spermatogenesis for their function in Sertoli cells [21,22], none of them have been described as involved during spermiogenesis.

Here, we show that *dLkb1* lethality can be fully rescued by overexpression of its human homologue. However, such rescued males are completely sterile indicating that dLKB1 is required for male fertility. *dLkb*1 is required in the germline for spermiation and we identified a testis-specific transcript, like for mammalian LKB1. In *Drosophila*, LKB1 is required for actin cone formation. Moreover, we found that some LKB1 targets are also involved in the germline in spermatid individualization, but none of them at the early step that requires LKB1. Finally, LKB1 accumulates concomitantly with myosin V along the flat side of elongating spermatid nuclei before actin cone formation. However, LKB1 and myosin V do not affect each other’s localization, although they are both required very early for actin cone formation.

## Results

### Human LKB1 rescues lethality but not male sterility of dLkb1 null mutants

Lethality of two *dLkb1* alleles (*Lkb1*^*4A4-2*^ and L*kb1*^*X5*^) can be rescued with *Drosophila dLkb1* transgenes driven either by the endogenous promoter (dLkb1>GFP-dLkb1) or by the ubiquitin promoter (Ubi>GFP-dLkb1) (called hereafter dLkb1-rescued males) (Fig 1B). Lethality can be rescued also by a human LKB1 cDNA fused to GFP and driven by the ubiquitin promoter (Ubi>GFP-hLKB1). However, while fly females were fertile after rescue by either *Drosophila* or human LKB1 transgenes, only dLkb1-rescued males were fertile. Males rescued with human LKB1 (called hereafter hLKB1-rescued males) were fully sterile (Fig 1B).

Analysis of testes from wild type (WT) and hLKB1-rescued males using DNA and F-actin staining showed that seminal vesicles of control males were swollen and filled with many spermatozoa, as indicated by the elongated sperm nuclei that were independent from each other (Fig 1C). Conversely, in testes from hLKB1-rescued males, seminal vesicles were flat and empty (Fig 1D). Moreover, many more nuclear bundles accumulated in the testis caudal part in hLKB1-rescued males compared with controls (59.5±3.0 vs 28.0±2.5, n = 12, p value <0.001) (Fig 1F and 1G), suggesting that sperm individualization was delayed or stopped in hLKB1-rescued flies. These observations suggest that hLKB1-rescued males do not produce mature individualized spermatozoa.

### LKB1 is required in the germline for spermatid individualization

The arrangement of the 64 spermatid nuclei, their morphological changes and elongation were comparable in testis squashes from WT and hLKB1-rescued males (Fig 1I and 1J). This suggests that the early stages of spermatogenesis occur normally in hLKB1-rescued males. As nuclear division, cyst elongation, and polarity appeared to be normal in hLKB1-rescued males, we focused on the post-elongation stages of spermatogenesis, particularly sperm individualization. We examined actin cone formation, which is the first step of individualization complex formation (schematic in Fig 1A), in whole or squashed testes from hLKB1-rescued and WT males by F-actin staining. We detected well-ordered arrays of F-actin cones (arrow in Fig 1I’) in individualization complexes in WT testes, but never in mutant testes (Fig 1J’). Moreover, in testes from hLKB1-rescued males, the overall distribution of F-actin was drastically changed, and abnormal clumps (arrowhead) of F-actin staining were present in the rear end of the nuclei (Fig 1J’).

**Fig 2:**
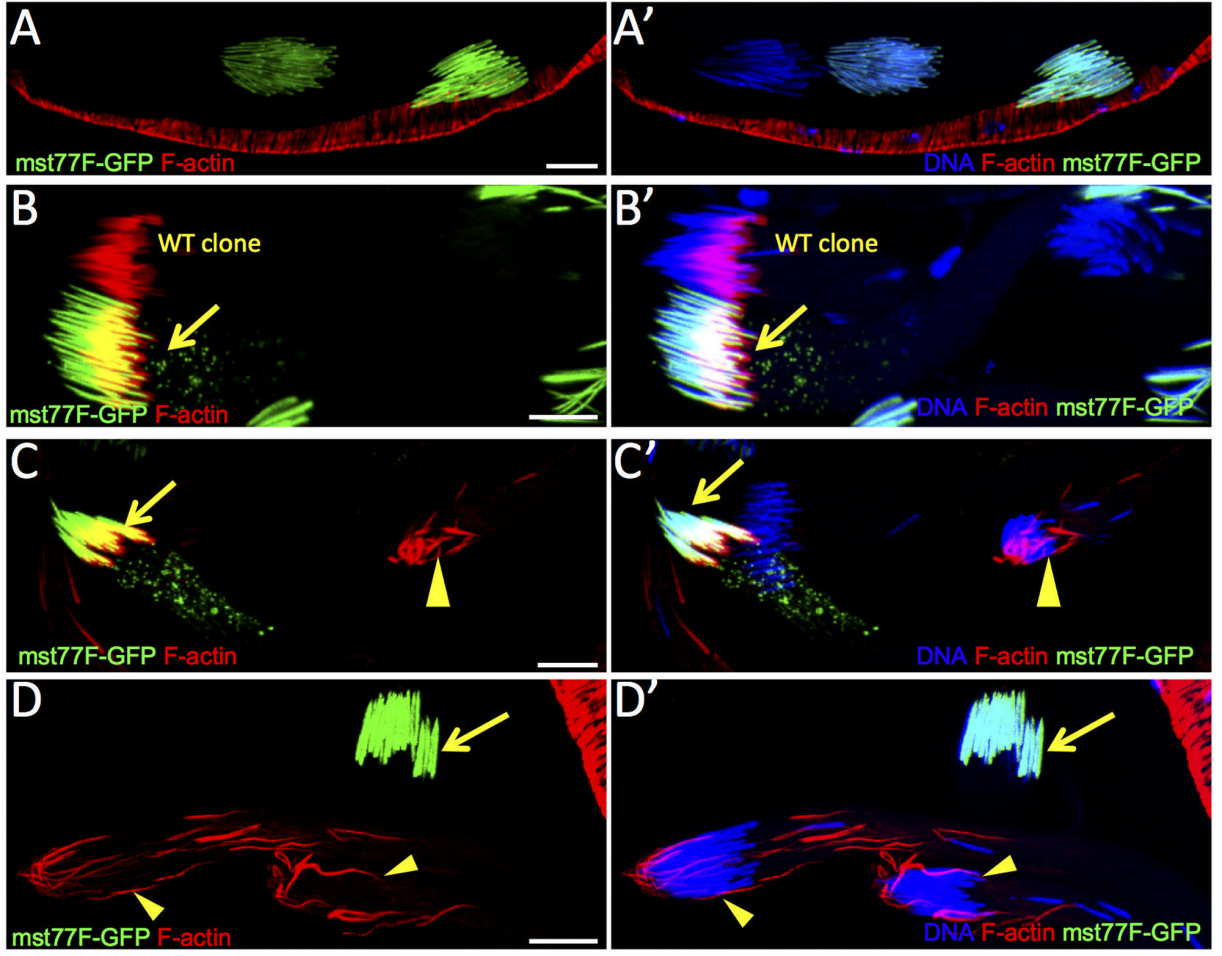
Generation of *dLkb1* mutant germline clones in testis germ cells. A-A’) Expression level of Mst77F-GFP fusion protein increases as spermatid nuclei become more elongated. B-B’) WT clones do not express GFP showing that Mst77F-GFP is a suitable marker of mitotic recombination. C-C’ and D-D’) *Lkb1*^x5^ mutant clones were generated and detected by the absence of GFP. Arrows indicate WT nuclei and arrowheads mutant clones. Scale bars = 10μm.

To confirm that sterility in hLKB1-rescued males was due to *dLkb1* loss of function, we performed two different approaches. First, we used *dLkb1* UAS-RNAi lines driven by bam-GAL4 to deplete dLKB1 specifically in germ cells. Note that, although bam-GAL4 is mainly expressed in spermatogonial cysts at the 8-16-cell stages, it is able to drive expression of siRNA to levels sufficient to severely deplete some proteins during individualization like myosin VI [23]. We obtained the same phenotype with three different RNAi lines (Table 1): empty seminal vesicles (Fig 1E), accumulation of 64-spermatid cysts in the testis caudal region (Fig 1H), normal formation of nuclear bundles (Fig 1K) and impaired formation of actin cones (Fig 1K’). Second, we generated mitotic recombination clones that lack *dLkb1* specifically in the germline. However, the GFP reporters normally used for such analysis are not expressed in elongated spermatids (not shown). Indeed, in *Drosophila*, transcription of most genes stops after the spermatocyte stage, upon meiotic division and before spermatid elongation [2]. mRNAs from genes required in the late steps of spermatogenesis are stored until spermatid morphogenesis, when they are translated after degradation of all the other transcripts [2]. Therefore, we took advantage of fusion proteins that encode the linker histone-like protein MST77F or protamine B fused to GFP to visualize the clones. These two fusion proteins localize in the nuclei of spermatids and are expressed only during late spermatogenesis [24]. Specifically, MST77F-GFP starts to accumulate in the elongating nuclei (early canoe stage) of spermatids well before actin cone formation (Fig 2A and 2A’). Moreover, also during actin cone formation and migration, nuclei retain some GFP expression. By producing clones of mitotic recombination between chromosome arm heterozygous for MST77F-GFP (or protamine B-GFP, not shown) transgenes, we could identify germline clones marked by the absence of GFP at different spermatid stages, before and during actin cone formation, as depicted with an example of a control clone (no associated mutation) (Fig 2B and 2B’). Homozygous *Lkb1*^*X5*^ mutant cysts were marked by the absence of MST77F-GFP expression, reduced F-actin, and clumps of F-actin associated with the rear end of nuclei with only few cone-like actin structures that never migrated (n = 23) (Fig 2C, 2C’, 2D and 2D’). This result confirms that LKB1 is required for spermiogenesis in *Drosophila*, and indicates that this effect is cell-autonomous in the germline.

**Table 1:** AMPK, KP78b and NUAK are involved in fly spermiogenesis.

Gene	CG number	RNAi line	Seminal vesicle	Male fertility	Actin cone formation	Actin cone migration
**lkb1**	CG9374	*KK*: *108356*	**Empty**	**Sterile**	**None**	Not relevant
		*TRiP*.*GL00019*	**Empty**	**Sterile**	**None**	Not relevant
		*TRiP*.*HMS01351*	**Empty**	**Sterile**	**None**	Not relevant
**AMPK**	CG3051	*KK*: *106200*	Full	Fertile	WT	WT
		*TRiP*.*HMS00362*	Full	Fertile	**Normal looking cones**	**uncoordinated migration of small cones**
		*NIG*.*3051R-1*	Full	Fertile		
		*NIG*.*3051R-2*	Full	Fertile		
**BRSK1**	CG6114	*GD*: *22224*	Full	Fertile	WT	WT
		*KK*: *100717*	Full	Fertile	WT	WT
**SIK2**	CG4290—	*GD*: *26496*	Full	Fertile	WT	WT
		*GD*: *26497*	Full	Fertile	WT	WT
		*KK*: *103739*	Full	Fertile	WT	WT
**PAR-1**	CG8201	*GD*: *52553*	Full	Fertile	WT	WT
		*GD*: *52556*	Full	Fertile	WT	WT
**SIK3**	CG15072	*GD*: *39864*	Full	Fertile	WT	WT
		*GD*: *39866*	Full	Fertile	WT	WT
		*KK*: *107458*	Full	Fertile	WT	WT
		*TRiP*.*JF03002*	Full	Fertile	WT	WT
**NUAK**	CG11870	*GD*: *16334*	**0–10**	**Semisterile**	**Two parallel streaks of actin**	**streaks of actin, uncoordinated migration**
		*KK*: *106088*	**0–10**	**Semisterile**		
		CG11871R-1	**0–10**	**Semisterile**		
		*TRiP*.*JF02162*	Full	Fertile	WT	WT
**KP78a**	CG6715	*GD*: *47658*	Full	Fertile	WT	WT
		*GD*: *51616*	Full	Fertile	WT	WT
		*NIG*.*6715R-3*	Full	Fertile	WT	WT
		*NIG*.*6715R-7*	Full	Fertile	WT	WT
**KP78b**	CG17216	*GD*: *51995*	Full	Fertile	WT	WT
		*GD*: *51996*	Full	Fertile	**Normal looking cones**	**uncoordinated migration of thin cones**
		*KK*: *105265*	Full	Fertile		
		*TRiP*.*JF02169*	Full	Fertile		
**SNRK**	CG8485	*GD*: *35939*	Full	Fertile	WT	WT
		*GD*: *35940*	Full	Fertile	WT	WT

The table shows the bam-GAL4–driven UAS RNAi lines against AMPK-related kinases used in this study. The line origin and the seminal vesicle, male fertility, actin cone formation and actin cone migration phenotypes are indicated for each line. WT, wild type. At least 30 males were analysed for each genotype.

### A testis-specific transcript of *Drosophila* LKB1

**Fig 3:**
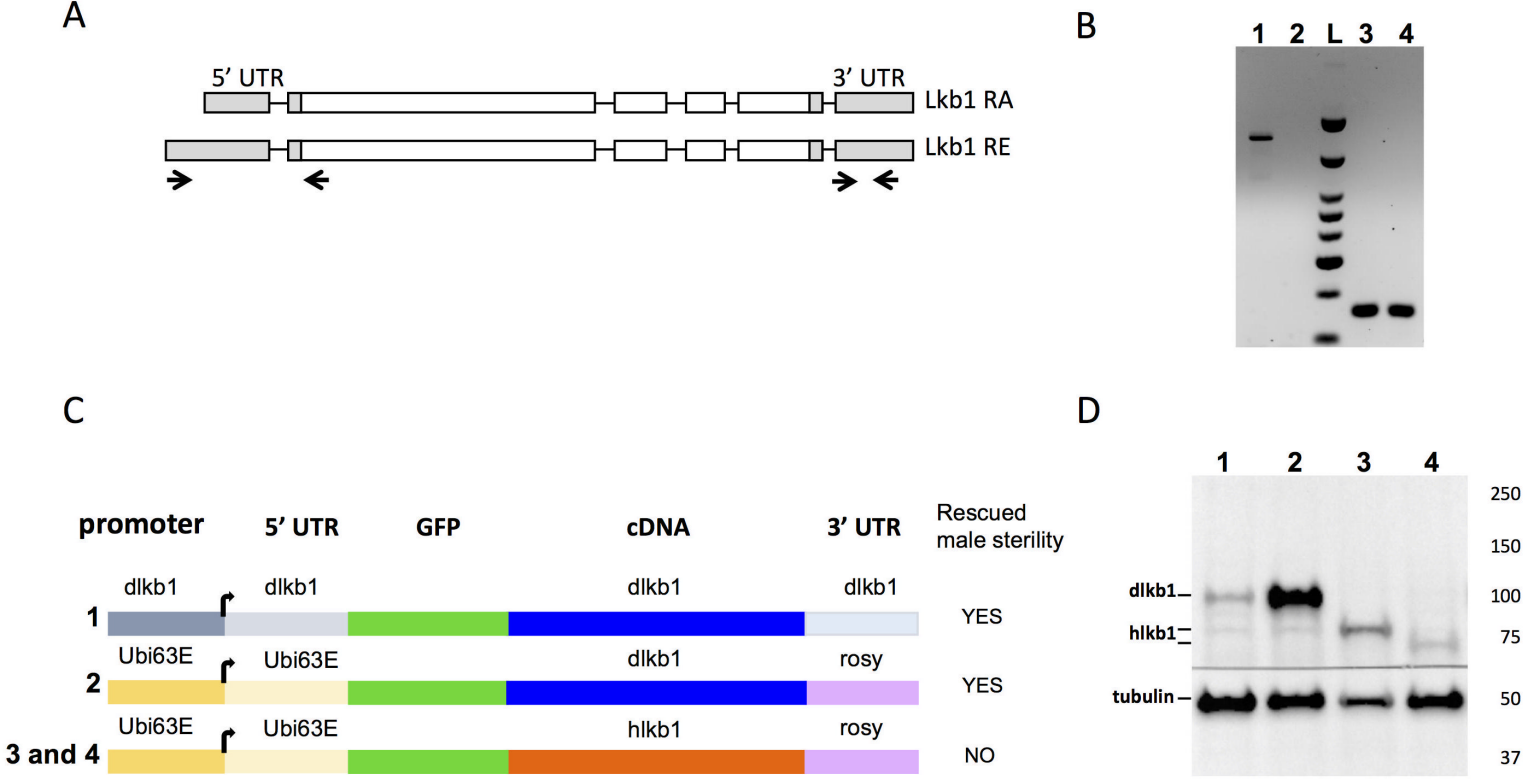
Analysis of endogenous and transgene LKB1 expression in the testis. **(A)** Schematic representation of the two different mRNAs of the *Drosophila Lkb1* gene. Primers used for the RT-PCR assay shown in B are indicated below with arrows: a, primers in the 5’UTR and b, primers in the 3’ UTR. The primers in the first exons amplify only a male-specific transcript (amplicon of 592bp), while the primers in the 3’UTR amplify all transcripts (amplicon of 131bp). **(B)** RT-PCR assay with primers that amplify the 5’UTR (a) or the 3’ UTR (b). T: testis RNA; O: ovary RNAs. **(C)** Schematic representation of the different transgenes that express dLkb1 or hLKB1. Numbers on the left correspond to the lanes on the western blot shown in D. On the right column is indicated whether the transgene can rescue male sterility. **(D)** Testis protein extracts from adult flies that express dLkb1 or hLKB1 were analyzed by western blotting with an anti-GFP antibody. The lane numbers correspond to the transgenic lines shown in C: 1) dLkb1>dLkb1-GFP, 2) Ubi>dLkb1-GFP, 3) Ubi>hLKB1L-GFP, and 4) Ubi>hLKB1S-GFP. The anti-tubulin antibody was used as loading control.

It was previously reported that there are two mouse and human LKB1 isoforms resulting from alternative splicing of the last exon: the short LKB1S isoform that is testis-specific, and the long LKB1L isoform that is expressed in all tissues [18,19]. However, like Ubi>GFP-hLKB1, the Ubi>GFP-hLKB1S transgene could not rescue male fertility in *Drosophila* carrying the *Lkb1* mutant alleles (Fig 3C). It suggests that LKB1S isoform does not have any testis-specific and conserved feature that could explain the absence of sterility rescue by LKB1 long isoform in fly.

Using Flybase GBrowse, we noticed the presence of two types of dLkb1 ESTs with different 5’UTRs: a type with a long 5’UTR that was only present in testis ESTs (12 ESTs), and a type with a short 5’UTR present in all tissues and at all stages (more than 100 ESTs). Both RNA encode the same protein. RT-PCR analysis confirmed that the long transcript was expressed in the testis but not in the ovary (Fig 3A and 3B). Thus, *Drosophila* expressed a testis specific transcript of LKB1 as in mammals.

Western blot analysis of the expression of Ubi>GFP-dLkb1 and Ubi>GFP-hLKB1 using an anti-GFP antibody showed a larger accumulation of dLKB1 than of hLKB1 (both L and S isoforms) in testes of rescued males (Fig 3C and 3D). This difference could be explained by a higher instability of hLKB1 mRNA or protein. Moreover, although accumulation of hLKB1 protein from the Ubi>GFP-hLKB1 transgene (lanes 3 and 4 in Fig 3D) was comparable to that of dLKB1 from the dLkb1>GFP-dLkb1 transgene (lane 1 in Fig 3D), this was not sufficient to rescue male sterility (Fig 3C). Thus, these results suggest that either dLKB1 owns a molecular function that is not shared with hLKB1, or the weaker stability of hLKB1 could be limiting for the rescue of sterility.

### Three AMPK-related kinases are involved in spermatogenesis, but none as early as LKB1

**Fig 4:**
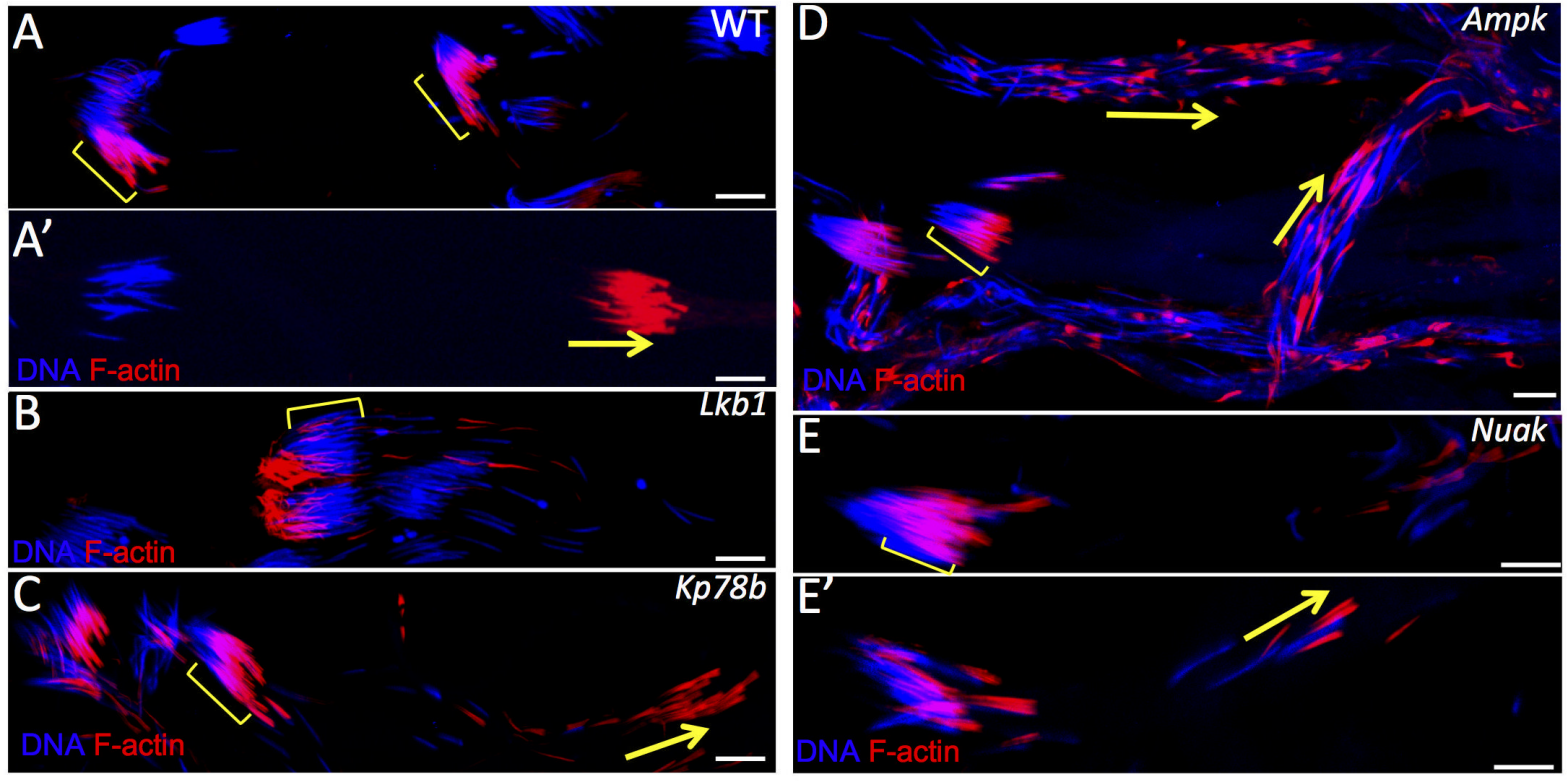
The AMPK-related kinases AMPK, KP78b and NUAK are involved in actin cone shaping and migration. Representative images of actin cone formation and migration in spermatid bundles from wild type (WT) (A and A’), or from the following bam-GAL4–driven UAS RNAi lines: (B) *Lkb1*, (C) *Kp78b*, (D) *Ampk* and (E and E’) *Nuak*. Arrows indicate migrating actin cones; brackets indicate formation of actin cones on nuclear bundles. Scale bars = 10μm.

LKB1 functions as a master protein kinase that regulates AMPK and AMPK-related kinases [10,11]. Nine such kinases are present in the fly genome and we tested 2 to 4 different RNAi lines (Table 1) (n>30 per genotype) to attenuate the expression of each of them. These UAS-RNAi transgenes were driven by bam-GAL4, as previously done for *dLkb1* RNAi. After RNAi-mediated downregulation of each kinase, we analyzed actin cone formation and migration in testis squashes (Table 1). Actin cones formed at the level of the bundle of 64 spermatid nuclei and migrated in a coordinated manner and tightly packed in WT (Fig 4A), but not in *dLkb1* RNAi mutants (Fig 4B) where extra F-actin accumulates at the rear end of the nuclei. In the RNAi lines (3 out of 4) for *Kp78b*, an AMPK-related kinase with no clear orthologue in mammals, actin cones formed correctly but their migration was uncoordinated and they appeared smaller than in WT (Table 1, Fig 4C). Similarly, in the *Ampk* RNAi lines (3 out of 4), actin cones formed correctly, but then became disorganized and migrated individually and remained often associated with their nuclei (Table 1, Fig 4D). However, in both knockdowns, seminal vesicles contained a lot of free spermatozoa and these males were fully fertile, indicating that actin cone shape modification and uncoordinated migration do not totally abolish spermiation (Table 1). In the *Nuak* RNAi lines (3 out of 4), actin cones started to form, but the cone shape was affected (no base forming, but presence of two parallel actin streaks) and cone migration was uncoordinated and disorganized (Table 1 and Fig 4E). These males had seminal vesicles with very few spermatozoa and were almost sterile (0 to 10% of progeny compared with WT males) (Table 1). RNAi-mediated downregulation of all the other genes encoding AMPK and AMPK-related kinases did not affect fertility, spermatogenesis or actin cone formation. This suggests that they play no role in spermatid individualization, although it cannot be formally excluded that the phenotypes induced by these RNAi transgenes are not identical to those of genetic null mutants.

In conclusion, these experiments show that although AMPK, NUAK and KP78b play a role in spermatid individualization, their downregulation does not affect actin cone formation as early as in *dLkb1* mutants. This could be due to redundancy of these three genes, a hypothesis reinforced by the fact that AMPK, NUAK and KP78b belong to the same protein kinase family and may have common targets. Alternatively, the *Lkb1* phenotype could be due to the sum of the individual and distinct effects of the three kinases. However, the analysis of the double RNAi lines and one triple RNAi line (Table 2) for these three genes did not highlight any additional change in the phenotype of actin cone formation. This indicates that they do not block actin cone formation as early as *dLkb1* does. Therefore, although the hypomorphic nature of RNAi knock-down does not allow us to formally exclude their involvement, these results suggest that LKB1 act on an alternative target at early stages of actin cone formation.

**Table 2:** RNAi combinations did not reveal any additive effect between AMPK-related kinases.

Gene	RNAi line	Seminal vesicle	Male fertility	Actin cone Phenotype
**NUAK**	*KK*: *106088*	**0–10**	**Semisterile**	**NUAK phenotype**
**AMPK**	*TRiP*.*JF01951*			
**NUAK**	*KK*: *106088*	**0–10**	**Semisterile**	**NUAK phenotype**
**AMPK**	*TRiP*.*HMS00362*			
**NUAK**	*KK*: *106088*	**0–10**	**Semisterile**	**NUAK phenotype**
**KP78b**	*TRiP*.*JF02169*			
**AMPK**	*NIG*.*3051R-1*	>200	Fertile	**AMPK phenotype**
**KP78b**	*TRiP*.*JF02169*			
**KP78b**	*KK*: *105265*	>200	Fertile	**AMPK phenotype**
**AMPK**	*TRiP*.*JF01951*			
**NUAK**	*KK*: *106088*	**0–10**	**Semisterile**	**NUAK phenotype**
**AMPK**	*NIG*.*3051R-1*			
**KP78b**	TRiP.JF02169			

The table lists the bam-GAL4–driven UAS RNAi lines used to make double RNAi or triple RNAi mutants for the AMPK-related kinases NUAK, AMPK and KP78b. The seminal vesicle, male fertility, and actin cone phenotypes are indicated for each combination. At least 30 males were analysed for each genotype.

### LKB1 localizes at spermatid nuclei before actin cone formation

**Fig 5:**
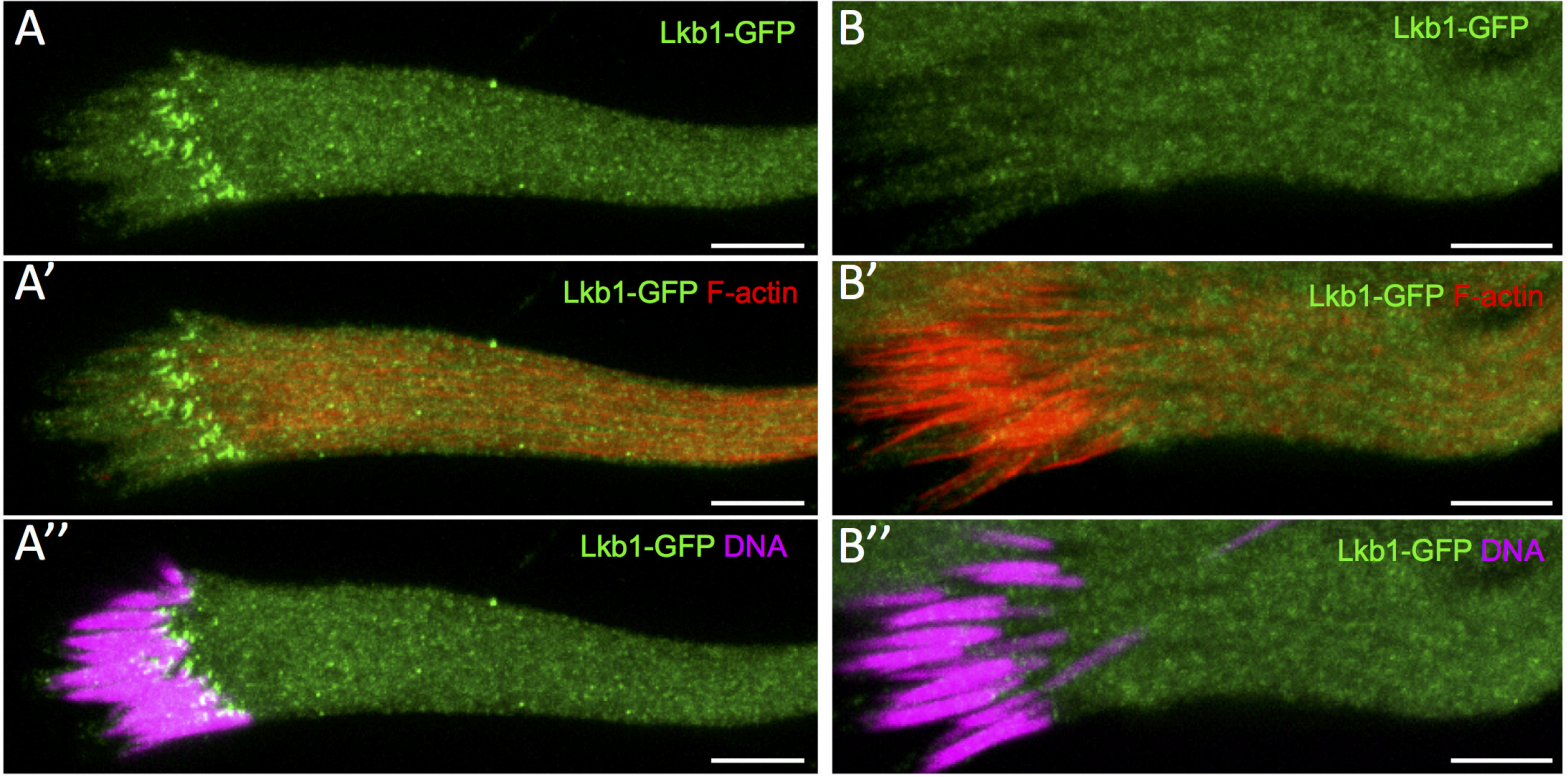
LKB1 accumulates at the front side of the WT spermatid nuclei before actin cones become detectable. Isolated pre-individualization cysts stained with Hoechst (DNA), phalloidin (F-actin) and an anti-GFP antibody to detect dLkb1-GFP. Lkb1-GFP strongly accumulates asymmetrically at the front side of the WT nuclei of elongating spermatids (A, A”) before any actin cone becomes detectable (A’). Lkb1-GFP signal has disappeared (B, B”) when nuclei are fully elongated and actin cones are forming (B’). Scale bars = 10μm.

To better understand LKB1 role in spermiogenesis, we analyzed LKB1 subcellular localization in WT cysts. To this aim, we used the dLkb1>GFP-dLkb1 construct that rescued all the known *Lkb1* phenotypes [15]. LKB1 accumulated as small dots at the front end of every spermatid nucleus before the appearance of actin cones (Fig 5A, 5A’ and 5A”) and was absent when actin cones became visible (Fig 5B, 5B’ and 5B”). LKB1 accumulates on the side of the elongated spermatid nuclei that is close to the basal body.

**Fig 6:**
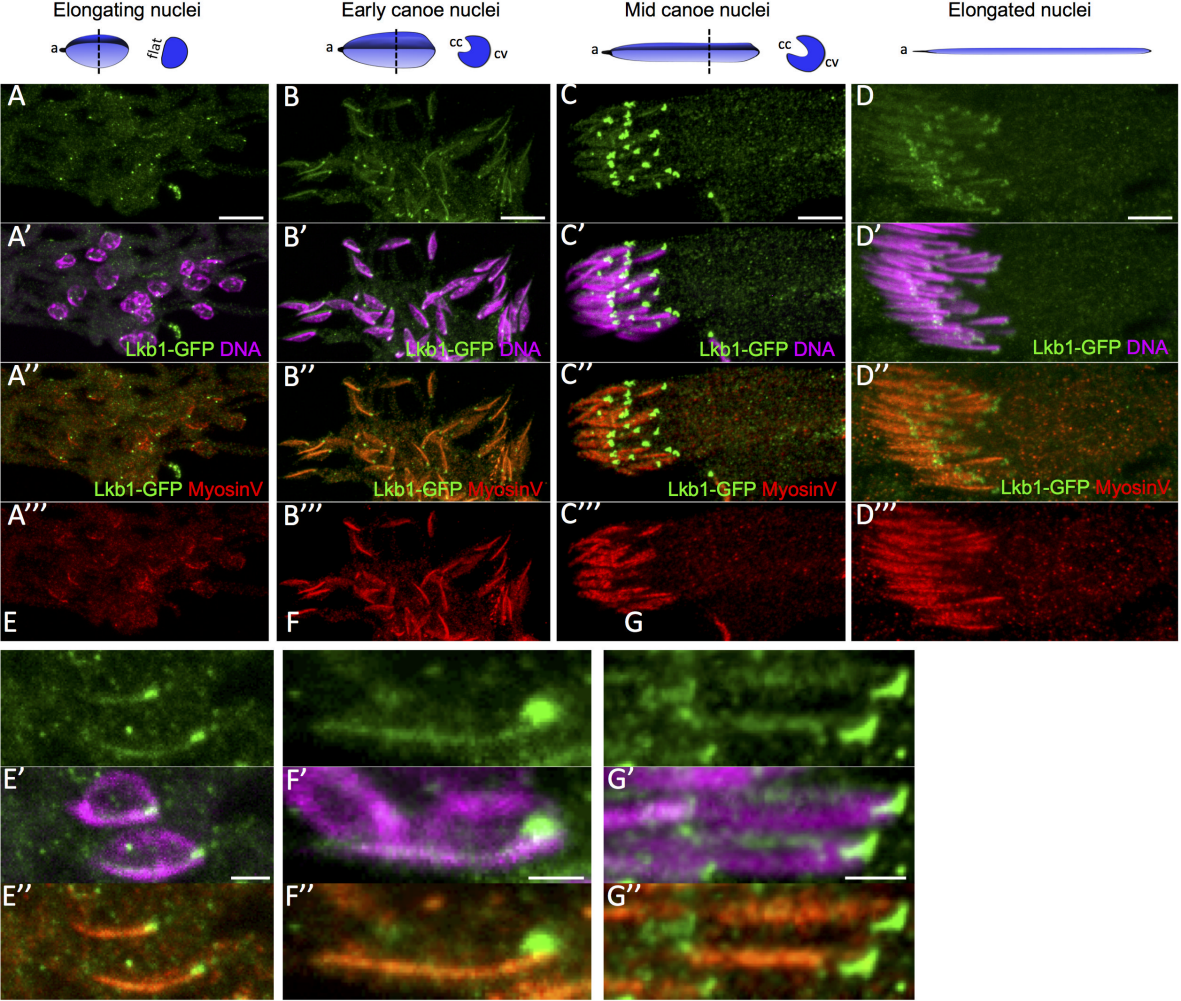
Dynamic LKB1 and myosin V expression and localization during WT spermatid nuclear shaping. Post-meiotic WT male germ cells in a cyst (A to D) were incubated with anti-GFP (dLkb1-GFP) and anti-myosin V antibodies and stained with Hoechst (DNA) at different stages: beginning of spermatid nucleus elongation (A), early (B), mid (C) and late canoe (D) stages. The shape of one full nuclei and a transverse section at the level of the dotted line is shown for each stage to indicate the acrosome (a), and the flat side or the convex (cv) and concave (cc) sides of the nuclei. E to G are higher magnifications showing one or two nuclei at the elongating (E), early canoe (F) and mid-canoe (G) stages. Lkb1-GFP and myosin V accumulates asymmetrically in elongating (A, E), early canoe (B,F), mid canoe (C,G) and elongated nuclei (D) of spermatids. (A to D) Scale bars = 10μm. (E to G) Scale bars = 2μm.

During spermatid maturation, the spermatid nucleus changes from spherical to needle-shaped with highly condensed chromatin (Fig 6). The spherical nucleus first flattens on the side where there is the highest chromatin concentration. Then, the flattened side becomes concave, giving a “canoe” shape to the nucleus. In parallel, chromatin accumulates at the opposite site of the cavity [1,2]. From the beginning of the elongation of spermatid nuclei, we could detect LKB1 on the flat part of nuclei where most of the chromatin accumulated (Fig 6A and 6E). During the early canoe stage, LKB1 strongly accumulated at the front end of elongating nuclei (opposite to the acrosome) and was present as a very faint line on the concave side where chromatin was still enriched (Fig 6B and 6F). At later canoe stages, when DNA was concentrated on the convex side, LKB1 remained associated with the front tip of nuclei, close to the concave side and was still present as a faint streak on the flat side (Fig 6C and 6G). When chromatin was nearly fully condensed, shortly before the onset of sperm individualization, LKB1 intensity is strongly reduced from the nuclei (Fig 6D) to eventually disappear (data not shown). Thus, LKB1 starts to be expressed very early during the elongation of spermatid nuclei before they are aligned in parallel bundles, and shows a dynamic and asymmetric localization pattern.

### Myosin V accumulates at spermatid nuclei at the same time as LKB1

In fly testes, myosin V, which is encoded by the *didum* gene, localizes to the narrow terminal ends of spermatid nuclei, while individualization complexes are forming, and is lost when individualization complexes begin to migrate [7]. Moreover, individualization complexes in *didum*^*Q1052st*^ mutant testes are severely disrupted [7]. As myosin V localization and loss of function phenotype are similar to those we observed for LKB1, we reanalyzed myosin V localization and function before spermatid individualization.

Using an antibody against myosin V, we found that it started to accumulate as a faint band along the flattened side of spermatid nuclei at the time of LKB1 localization (Fig 6A”, 6A”’, 6B”, 6B”’, 6E and 6F). At the canoe stage, myosin V accumulated on the flattened side of spermatid nuclei, but not at its most front tip where LKB1 was present (Fig 6C” and 6G). Eventually, myosin V expression decreased after late canoe stage nuclei (Fig 6D” and 6D”’) and disappeared when nuclei were fully elongated and actin cones became visible. These findings indicate that myosin V accumulates asymmetrically on spermatid nuclei before any actin cone can be detected, which is earlier than it has been described so far [7].

**Fig 7:**
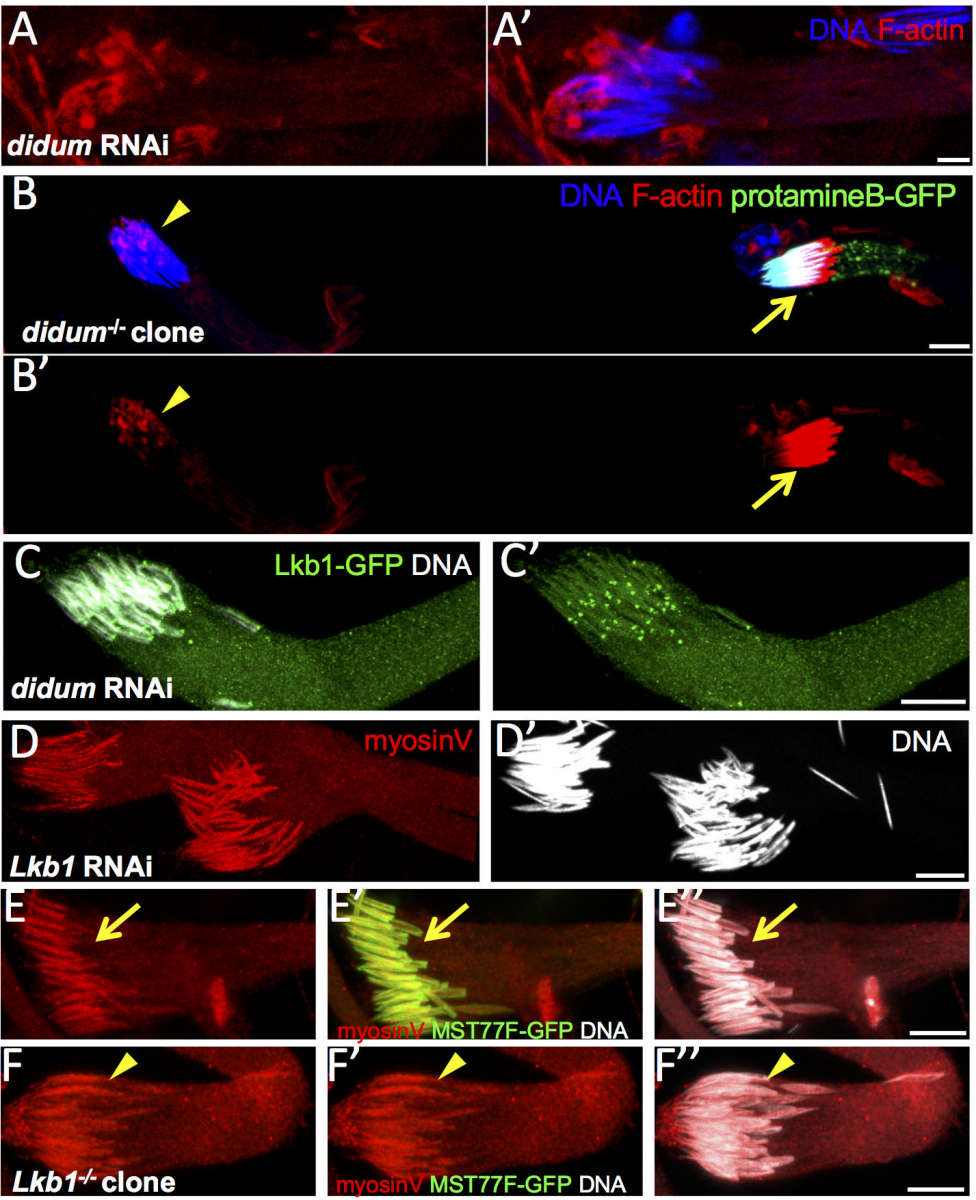
Myosin V loss of function mimics LKB1 loss of function. A) Spermatid nuclei from bam-GAL4–driven *didum* (myosin V) RNAi flies were stained with phalloidin (F-actin, red) and Hoechst (DNA, blue). B-B’) *didum*^*154*^ mutant clones are detected by the absence of protamine B-GFP expression. Arrows, WT bundle of nuclei; arrowheads, mutant clones. Upon myosin V loss of function, no actin cone is formed. C) Spermatid cells from *didum* RNAi clones incubated with an anti-GFP antibody (Lkb1-GFP, green) and stained with Hoechst (DNA, white). D) *Lkb1* RNAi does not prevent myosin V (red) accumulation in spermatid nuclei (Hoechst, white). E, F) Differently from wild type (E), Lkb1^x5/x5^ germline clones, which are identified by the lack of MST77F-GFP expression (green), accumulate myosin V (red) in spermatid nuclei (white). Arrows, WT bundles of nuclei; arrowheads, mutant clones. Scale bars = 10μm.

Then, we depleted myosin V in the male germline using RNAi against *didum*. These males were sterile and actin cone formation was severely disrupted with almost no cone forming and accumulation of actin clumps close to the nuclei (Fig 7A and 7A’). Similarly, in *didum* null mutant clones, no actin cone formed on elongated nuclei bundles, with only few actin clumps (Fig 7B and 7B’). These phenotypes were similar to those previously described using transheterozygous mutant flies [7], and were strongly reminiscent of the *dLkb1* loss of function phenotype. They also confirmed that myosin V function is required in the germline for spermatid individualization.

However, RNAi-mediated depletion of myosin V in elongating spermatids had no effect on LKB1 accumulation (Fig 7C and 7C’). Similarly, after RNAi-mediated *dLkb1* downregulation, myosin V accumulated and correctly localized at spermatid nuclei from the beginning to the end of elongation (Fig 7D and 7D’). Myosin V expression was comparable also in WT and homozygous *Lkb1*^*X5*^ mutant cysts (Fig 7E and 7F). Thus, although both LKB1 and myosin V are required for the initiation of actin cone formation and are concomitantly expressed during spermatid nucleus elongation, but neither is dependent on the other for proper localization.

## Discussion

Our work reveals that in *Drosophila*, LKB1 is required in the male germ line for sperm individualization and identified a testis-specific transcript of dLKB1, as previously reported in mice [18,19]. To our knowledge, LKB1 is the first example of a gene with a conserved requirement in sperm individualization. Thus, whereas the process of individualization and cytoplasm excess elimination seems very different between fly and mammals, our results suggest that this process may rely on molecular actors and mechanisms more conserved throughout evolution than previously expected.

Our results do not allow to clearly determine why hLKB1 is not able to rescue male sterility. A potential explanation is that hLKB1 is not produced at the right time and/or in the right place. In male germ cells, most transcription stops upon meiosis entry and some mRNAs are translationally repressed and stored to be translated later during spermiogenesis. The identification of a testis-specific dLKB1 transcript could reflects a requirement for a post-transcriptional regulation. Indeed, this transcript differs from the ubiquitous variant only by the presence of a longer 5’UTR, and is detected only in testes. These findings are in agreement with previous studies showing that the temporal and spatial regulations of the protamine B and *Mst77* gene expression involve the 5’ UTR and sequences contained in the coding region [25]. Moreover, dLKB1 is detected and required only during the late stages of spermatogenesis. However, the sterility rescue by Ubi-dLKB1 could argue against this idea. Nonetheless, the massive overexpression induced by this promoter and the higher dLKB1 protein stability compared to hLKB1 might compensate for an absence of post-transcriptional regulation. In the mouse, functional analysis of LKB1S and LKB1L proteins did not reveal any clear difference [19). Interestingly, LKB1S and LKB1L also differ by their 3’UTR. In mammals, spermiogenesis also relies on stored mRNA at early stages and their expression regulation usually depends on their 3’UTR, as exemplified, again, by the protamine genes [26,27]. Moreover, LKB1S is mainly detected at late stages of mouse spermatogenesis, days after the arrest of transcription [18,20]. Therefore, the possibility that, both in fly and in mammals, a testis-specific LKB1 transcripts allow a tissue-specific post-transcriptional regulation of their expression during the late steps of spermatogenesis would be an interesting hypothesis to explore.

LKB1 is the main upstream kinase responsible for the activation of 13 AMPK-related kinases in the mouse and nine similar kinases are present in the *Drosophila* genome [10]. They are the only known LKB1 targets. Here, we found that the reduction of function of three of them has a visible effect on spermatid individualization during *Drosophila* spermatogenesis, but at later steps than LKB1. AMPK, NUAK and KP78b absence affect actin cone shaping and stability and/or coordinated migration, but not the initial formation of actin cones, differently from *dLkb1* mutants. As these three AMPK-related kinases affect, although in different ways, actin cone migration, LKB1 could also be involved in the coordination of actin cone shaping and/or migration. However, the strong phenotype observed in actin cone formation initiation precludes the analysis of *dLkb1* function at later stages. Moreover, even the combined loss of function of these three kinases did not mimic *Lkb1* phenotype. Therefore, it is likely that LKB1 targets a yet-unknown molecule for its function in testis, though it cannot be excluded that this function is independent of its kinase activity.

In *Drosophila*, sterile mutants in which spermatogenesis proceeds to the assembly of highly elongated cysts, but fails to individualize spermatids have been previously described [5,6]. However, only in few of them, actin cone formation is blocked as observed in *Lkb1* and *didum* mutants. Some mutants in which nuclear shaping is altered also prevent actin cone formation, consistent with the hypothesis that spermatid nuclear membrane provide physical scaffolding for individualization complex assembly [28]. It has been observed that the actin filaments parallel to the elongated nuclei of *Drosophila* spermatids are primarily oriented with the barbed ends facing toward the membrane that surround the rear of the forming cones (Fig 1A”) [29]. Myosin V is the prototypical transport myosin specialized in the directed transport of cargoes toward actin filament barbed ends and in actin filament organization [30]. Myosin V could play a role in organizing the actin filaments on the nucleus side where it localizes. For instance, it could be anchored to the nucleus flat side where it could pull actin cables in the barbed-end direction toward the nucleus rear end to start actin cone formation.

Although their depletion leads to the same phenotype, LKB1 and myosin V localization do not depend on each other’s activity. Myosin V might play a role in physically organizing actin filaments. LKB1 asymmetric localization at the front end of the elongated spermatid nuclei suggests that it might give a polarity cue for the initiation of actin cone formation and for orienting their growth. However, PAR-1 and BRSK (encoded by *ssf* gene in fly), the two LKB1 targets with instructive roles for cell polarity in other systems, are not required in *Drosophila* testis. Additional work is necessary to define LKB1 molecular function in testis and identify its targets.

*Drosophila* sperm development has many parallels to spermiogenesis in other organisms, including mammals. In mice, LKB1 has a crucial role in spermiogenesis and male fertility [18]. Spermatozoa of LKB1S mutant mice are non-motile and in many of them the residual body, the mammalian equivalent of the fly waste bag, is not completely absorbed [18,20]. Moreover, in these mice, spermatozoa are very rare, suggesting an individualization defect, like the one we observed in flies. However, it has never been demonstrated that LKB1 is required in a cell autonomous manner in the mouse germline, whereas a cell autonomous function in Sertoli cells has been described [21]. To our knowledge, LKB1 is the first example of a gene required at these steps of spermiogenesis in both species, which argues for a conserved germline-specific function. It would be interesting to determine whether NUAK, AMPK, myosin V or any other gene involved in fly spermatid individualization plays a similar role in the mammalian male germline. Hence, the molecular and cellular mechanisms uncovered by the study of *Drosophila* spermiogenesis will enhance our understanding of similar processes, which are still poorly understood but crucial to human male fertility.

## Materials and methods

### *Drosophila* stocks and genetics

All fly stocks were maintained on standard *Drosophila* cornmeal agar and at 25°C.

Stocks were obtained from the Bloomington Drosophila Stock Center, unless indicated elsewhere. The RNAi lines GD and KK were obtained from VDRC [31], the NIG RNAi lines from the NIG-FLY facility, Japanese National Institute of Genetics, and the Trip RNAi lines from Harvard Medical School [32]. *Lkb1*^*4A4-2*^ and L*kb1*^*X5*^ are internal deletions at the LKB1 locus, both behaving as null mutant alleles [15,33]. RNAi in the male germline was induced using the bam-GAL4 driver, given by D. McKearin. dLKB1>GFP-dLKB1 transgene is gift of D St Johnston [15].

For mosaic analysis, protamine B-GFP and Mst77F-GFP strains [24] were recombined with the appropriate FRT chromosome using the original FLP/FRT method [34]. The original P {Mst77F-GFP} was jumped from the third chromosome to the right arm of the second chromosome. Mutant mosaic clones were induced in 1–2 day old males (*y*, *w*, hs:flp; FRT82B, Mst77F-GFP / FRT82B, *Lkb1*^*4A4-*^2 or FRT82B, *Lkb1*^*X5*^ and *y*, *w*, hs:flp; FRT42B, P{ProtamineB-GFP}/ FRT42B, *didum*^*154*^ or FRT42B d*idum*^*KG0434*^, [35]) with the FRT- and FLP-mediated recombination system and a heat-shock pulse at 37°C for 60 min, 9–10 days before dissection.

### Transgene cloning and expression monitoring

For LKB1 expression, the complete ORFs of *Drosophila* Lkb1 (dLkb1) and human LKB1 (hLKB1) were cloned in frame into a P-element based vector that contains the *Ubi-p63E* promoter and 5’UTR, the GFP coding sequence without stop codon, the Gateway cassette (Invitrogen) placed to allow fusion with the GFP in N-term position and the *rosy* 3’UTR with a polyadenylation signal [36]. Injection and transformant selection were performed by Bestgene. At least three independent insertions for all transgenes, excepted dlkb1>GFP-dLkb1 (one insertion), were analysed by western blot and for their rescue ability The results of the most representative are shown. Moreover, for the lines shown in the paper we checked by RT-qPCR on GFP sequence their expression in the testis and they are all in the same range (fold change <2), excepted the one with *lkb1* promoter, which was much weaker.

For western blotting, 2-day-old males (n = 15) were dissected in 50μl of lysis buffer, sonicated and boiled for 5 minutes. After centrifugation, 10μl of each supernatant was loaded on precast 4–15% acrylamide gels. After electrophoresis, proteins were transferred to nitrocellulose membranes (Biorad) and probed with mouse anti-GFP (dilution) (Ozyme, #JL-8) and anti-tubulin (1/10000) (Sigma #DM1A) antibodies.

### mRNA expression analysis

RT-PCR assays were performed using standard procedures. RNA was extracted from 30 pairs of testes or 10 pairs of ovaries. The RT reaction was performed using 500 ng total RNA, oligodT and the Superscript IV Reverse Transcriptase Kit (Invitrogen). The long dLkb1 transcript was amplified using primers in the 5’UTR (TTATTCCAGCGTTCGTCCCG and CCTCCATGGTGGTCACAGTC). Primers in the 3’ UTR (AGGAAATTCAGGCGCAACCT and ATAGCTTTCGTGTCGCTCCC) were used to amplify both long and short transcripts.

### Male fertility testing

To assess male fertility, each male (n = 20/genotype) was singly mated with five virgin wild type females at 25°C. The number of progeny was recorded and males were designated as sterile if mating resulted in no progeny, and as semi-sterile if the progeny number was less than 10% of that of controls (wild type mating).

### Immunostaining and imaging spermatid cysts

Testes from adult males were dissected in PBS and fixed in 4% paraformaldehyde in PBS for 16min followed by three washes in PBS. Testes were then partially opened with a tungsten needle. After blocking and permeabilization with PBS with 0.5% BSA and 0.1% Triton X-100 (PBT), testes were incubated with purified rabbit anti-myosin V (1/8000) [37] and goat anti-GFP (1/1000) (Abcam #5450) antibodies in PBT at 4°C overnight. After three washes in PBT, testes were incubated with the donkey anti–mouse Alexa Fluor 488 (Life Technologies) and donkey anti–rabbit Cy3 or Cy5 (Jackson ImmunoResearch Laboratories, Inc.) secondary antibodies (1/1,000 dilution) at RT for 2h.

Hoechst 33258 (Sigma Chemical) was used to stain DNA and Alexa568-phalloidin (Life Technologies) for F-actin. Stained specimens were mounted under coverslips using Antifade mounting medium (Vectashield, Vector Laboratories). Confocal images were acquired with a Leica SP5 confocal microscope. Images were cropped, rotated and adjusted for brightness and contrast with ImageJ.
